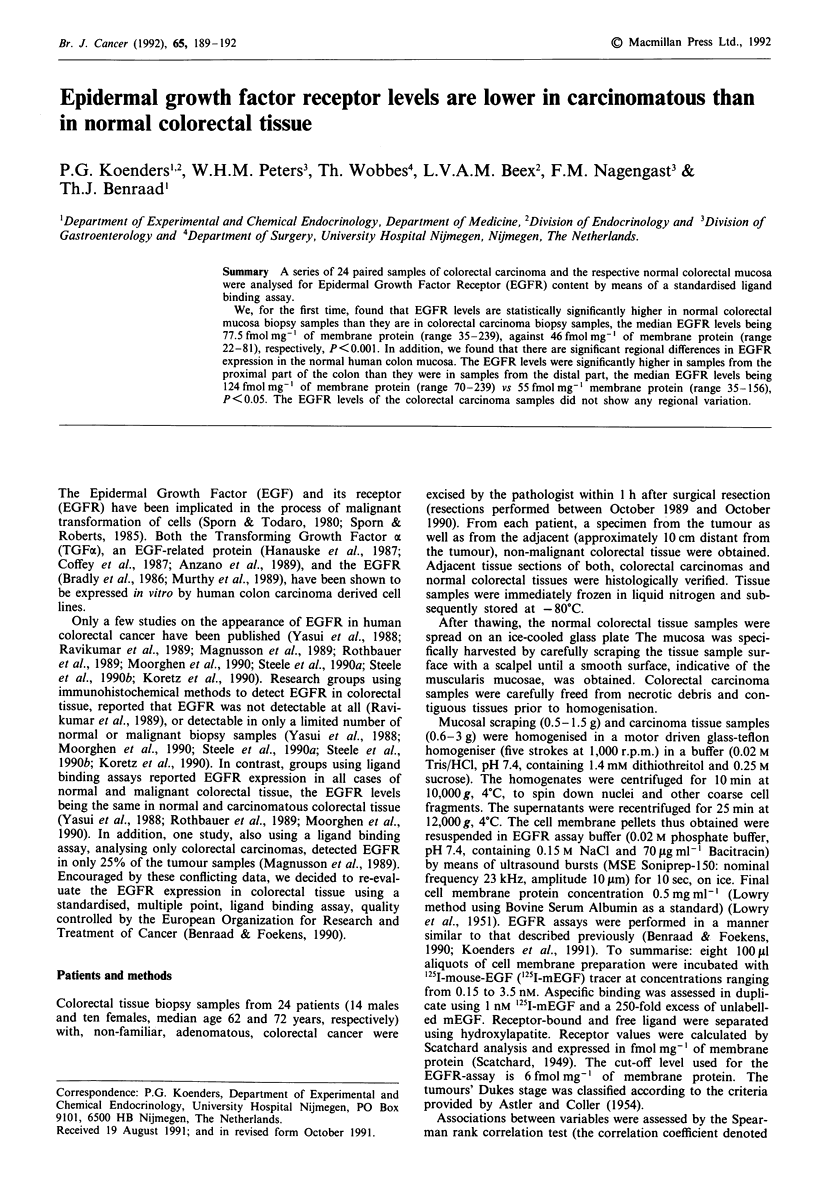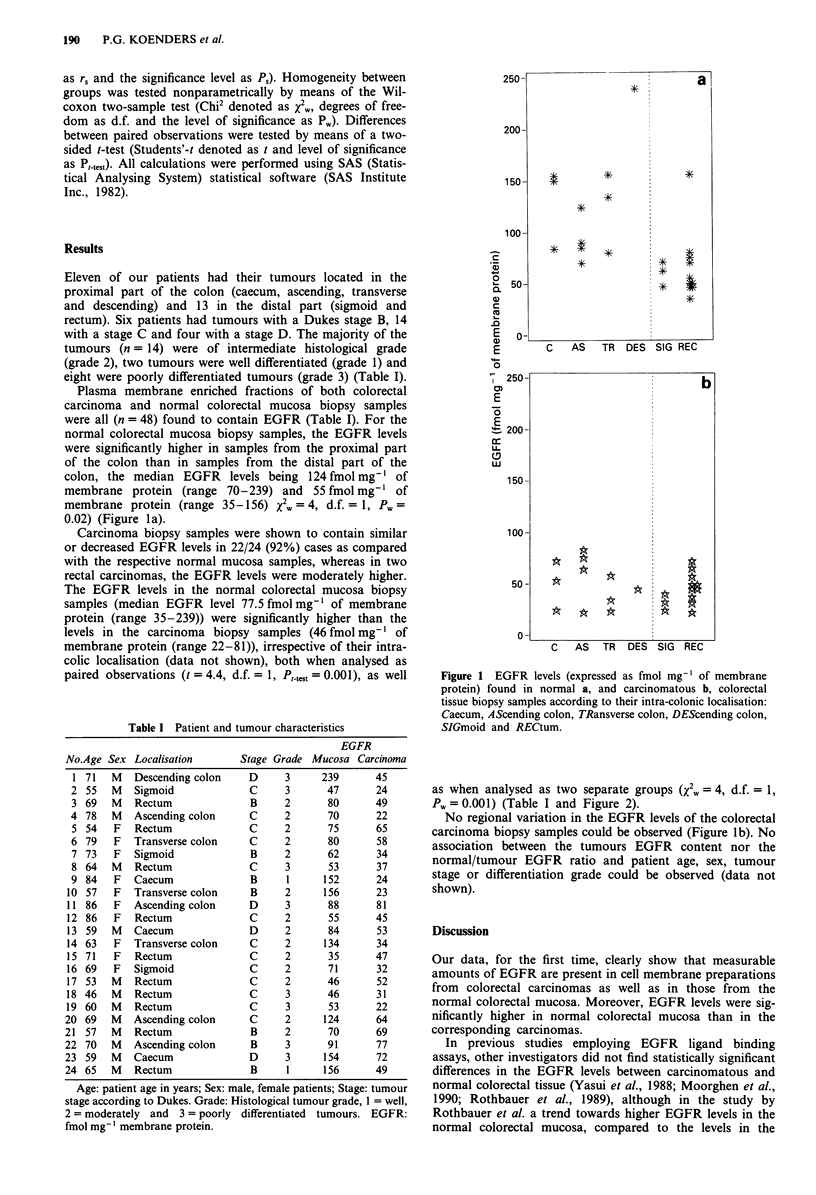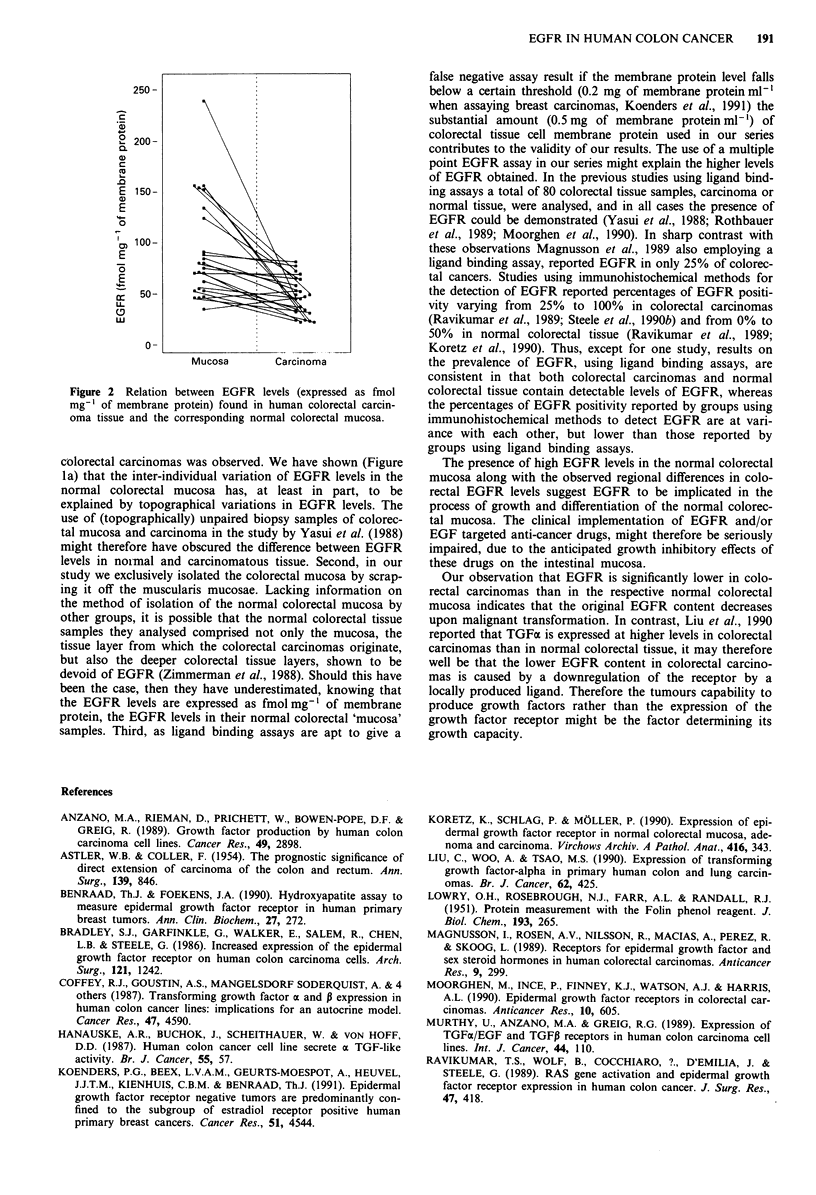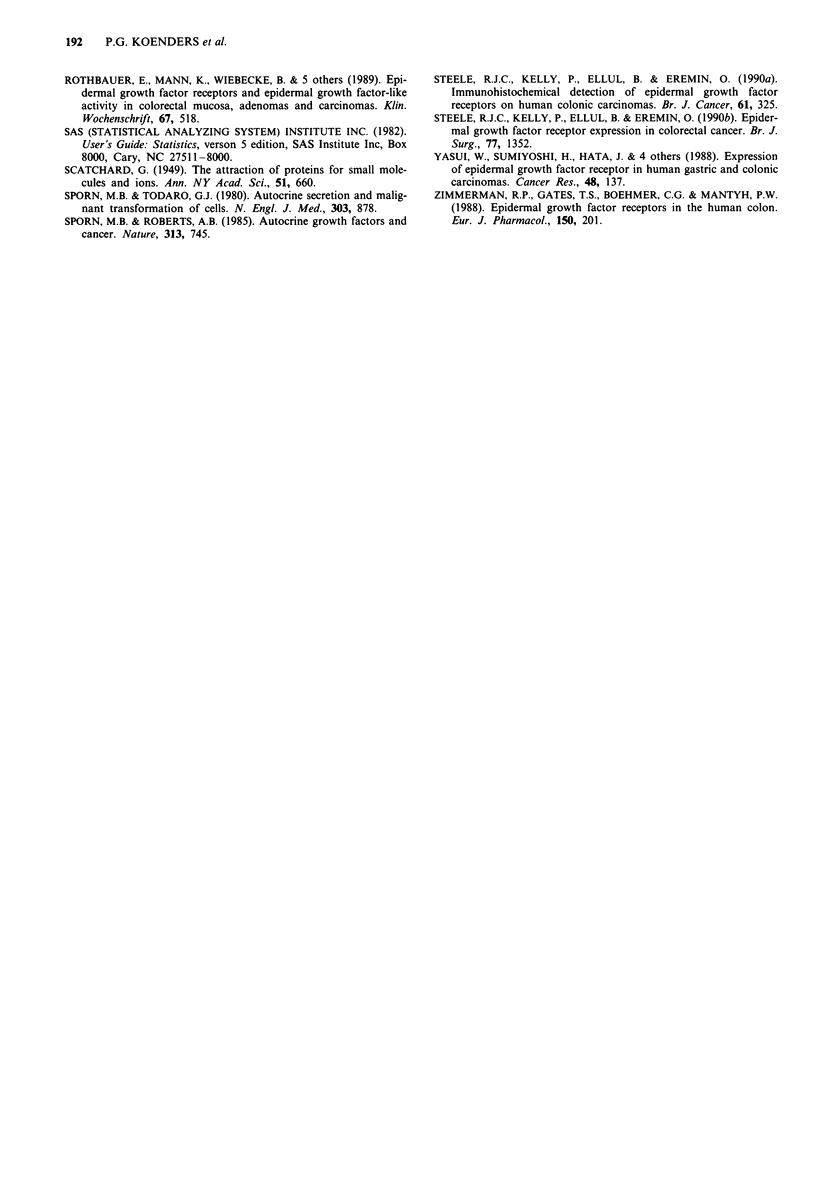# Epidermal growth factor receptor levels are lower in carcinomatous than in normal colorectal tissue.

**DOI:** 10.1038/bjc.1992.39

**Published:** 1992-02

**Authors:** P. G. Koenders, W. H. Peters, T. Wobbes, L. V. Beex, F. M. Nagengast, T. J. Benraad

**Affiliations:** Department of Experimental and Chemical Endocrinology, University Hospital Nijmegen, The Netherlands.

## Abstract

A series of 24 paired samples of colorectal carcinoma and the respective normal colorectal mucosa were analysed for Epidermal Growth Factor Receptor (EGFR) content by means of a standardised ligand binding assay. We, for the first time, found that EGFR levels are statistically significantly higher in normal colorectal mucosa biopsy samples than they are in colorectal carcinoma biopsy samples, the median EGFR levels being 77.5 fmol mg-1 of membrane protein (range 35-239), against 46 fmol mg-1 of membrane protein (range 22-81), respectively, P less than 0.001. In addition, we found that there are significant regional differences in EGFR expression in the normal human colon mucosa. The EGFR levels were significantly higher in samples from the proximal part of the colon than they were in samples from the distal part, the median EGFR levels being 124 fmol mg-1 of membrane protein (range 70-239) vs 55 fmol mg-1 membrane protein (range 35-156), P less than 0.05. The EGFR levels of the colorectal carcinoma samples did not show any regional variation.


Br. J. Cancer (1992), 65, 189-192 © Macmillan Press Ltd., 1992

### Epidermal growth factor receptor levels are lower in carcinomatous than in normal colorectal tissue

P.G. Koenders[1,2], W.H.M. Peters[3], Th. Wobbes[4], L.V.A.M. Beex[2], F.M. Nagengast[3] & Th.J. Benraad[1]

[1]*Department of Experimental and Chemical Endocrinology, Department of Medicine,* [2]*Division of Endocrinology and* [3]*Division of Gastroenterology and* [4]*Department of Surgery, University Hospital Nijmegen, Nijmegen, The Netherlands.*

**Summary** A series of 24 paired samples of colorectal carcinoma and the respective normal colorectal mucosa were analysed for Epidermal Growth Factor Receptor (EGFR) content by means of a standardised ligand binding assay.

We, for the first time, found that EGFR levels are statistically significantly higher in normal colorectal mucosa biopsy samples than they are in colorectal carcinoma biopsy samples, the median EGFR levels being 77.5 fmol mg$^{-1}$ of membrane protein (range 35–239), against 46 fmol mg$^{-1}$ of membrane protein (range 22–81), respectively, $P<0.001$. In addition, we found that there are significant regional differences in EGFR expression in the normal human colon mucosa. The EGFR levels were significantly higher in samples from the proximal part of the colon than they were in samples from the distal part, the median EGFR levels being 124 fmol mg$^{-1}$ of membrane protein (range 70–239) *vs* 55 fmol mg$^{-1}$ membrane protein (range 35–156), $P<0.05$. The EGFR levels of the colorectal carcinoma samples did not show any regional variation.

The Epidermal Growth Factor (EGF) and its receptor (EGFR) have been implicated in the process of malignant transformation of cells (Sporn & Todaro, 1980; Sporn & Roberts, 1985). Both the Transforming Growth Factor α (TGFα), an EGF-related protein (Hanauske *et al.*, 1987; Coffey *et al.*, 1987; Anzano *et al.*, 1989), and the EGFR (Bradly *et al.*, 1986; Murthy *et al.*, 1989), have been shown to be expressed *in vitro* by human colon carcinoma derived cell lines.

Only a few studies on the appearance of EGFR in human colorectal cancer have been published (Yasui *et al.*, 1988; Ravikumar *et al.*, 1989; Magnusson *et al.*, 1989; Rothbauer *et al.*, 1989; Moorghen *et al.*, 1990; Steele *et al.*, 1990*a*; Steele *et al.*, 1990*b*; Koretz *et al.*, 1990). Research groups using immunohistochemical methods to detect EGFR in colorectal tissue, reported that EGFR was not detectable at all (Ravikumar *et al.*, 1989), or detectable in only a limited number of normal or malignant biopsy samples (Yasui *et al.*, 1988; Moorghen *et al.*, 1990; Steele *et al.*, 1990*a*; Steele *et al.*, 1990*b*; Koretz *et al.*, 1990). In contrast, groups using ligand binding assays reported EGFR expression in all cases of normal and malignant colorectal tissue, the EGFR levels being the same in normal and carcinomatous colorectal tissue (Yasui *et al.*, 1988; Rothbauer *et al.*, 1989; Moorghen *et al.*, 1990). In addition, one study, also using a ligand binding assay, analysing only colorectal carcinomas, detected EGFR in only 25% of the tumour samples (Magnusson *et al.*, 1989). Encouraged by these conflicting data, we decided to re-evaluate the EGFR expression in colorectal tissue using a standardised, multiple point, ligand binding assay, quality controlled by the European Organization for Research and Treatment of Cancer (Benraad & Foekens, 1990).

### Patients and methods

Colorectal tissue biopsy samples from 24 patients (14 males and ten females, median age 62 and 72 years, respectively) with, non-familiar, adenomatous, colorectal cancer were excised by the pathologist within 1 h after surgical resection (resections performed between October 1989 and October 1990). From each patient, a specimen from the tumour as well as from the adjacent (approximately 10 cm distant from the tumour), non-malignant colorectal tissue were obtained. Adjacent tissue sections of both, colorectal carcinomas and normal colorectal tissues were histologically verified. Tissue samples were immediately frozen in liquid nitrogen and subsequently stored at −80°C.

After thawing, the normal colorectal tissue samples were spread on an ice-cooled glass plate The mucosa was specifically harvested by carefully scraping the tissue sample surface with a scalpel until a smooth surface, indicative of the muscularis mucosae, was obtained. Colorectal carcinoma samples were carefully freed from necrotic debris and contiguous tissues prior to homogenisation.

Mucosal scraping (0.5–1.5 g) and carcinoma tissue samples (0.6–3 g) were homogenised in a motor driven glass-teflon homogeniser (five strokes at 1,000 r.p.m.) in a buffer (0.02 M Tris/HCl, pH 7.4, containing 1.4 mM dithiothreitol and 0.25 M sucrose). The homogenates were centrifuged for 10 min at 10,000 *g*, 4°C, to spin down nuclei and other coarse cell fragments. The supernatants were recentrifuged for 25 min at 12,000 *g*, 4°C. The cell membrane pellets thus obtained were resuspended in EGFR assay buffer (0.02 M phosphate buffer, pH 7.4, containing 0.15 M NaCl and 70 μg ml$^{-1}$ Bacitracin) by means of ultrasound bursts (MSE Soniprep-150: nominal frequency 23 kHz, amplitude 10 μm) for 10 sec, on ice. Final cell membrane protein concentration 0.5 mg ml$^{-1}$ (Lowry method using Bovine Serum Albumin as a standard) (Lowry *et al.*, 1951). EGFR assays were performed in a manner similar to that described previously (Benraad & Foekens, 1990; Koenders *et al.*, 1991). To summarise: eight 100 μl aliquots of cell membrane preparation were incubated with $^{125}$I-mouse-EGF ($^{125}$I-mEGF) tracer at concentrations ranging from 0.15 to 3.5 nM. Aspecific binding was assessed in duplicate using 1 nM $^{125}$I-mEGF and a 250-fold excess of unlabelled mEGF. Receptor-bound and free ligand were separated using hydroxylapatite. Receptor values were calculated by Scatchard analysis and expressed in fmol mg$^{-1}$ of membrane protein (Scatchard, 1949). The cut-off level used for the EGFR-assay is 6 fmol mg$^{-1}$ of membrane protein. The tumours' Dukes stage was classified according to the criteria provided by Astler and Coller (1954).

Associations between variables were assessed by the Spearman rank correlation test (the correlation coefficient denoted

Correspondence: P.G. Koenders, Department of Experimental and Chemical Endocrinology, University Hospital Nijmegen, PO Box 9101, 6500 HB Nijmegen, The Netherlands.
Received 19 August 1991; and in revised form October 1991.

as $r_s$ and the significance level as $P_s$). Homogeneity between groups was tested nonparametrically by means of the Wilcoxon two-sample test (Chi$^2$ denoted as $\chi^2_w$, degrees of freedom as d.f. and the level of significance as $P_w$). Differences between paired observations were tested by means of a two-sided $t$-test (Students'-$t$ denoted as $t$ and level of significance as $P_{t\text{-test}}$). All calculations were performed using SAS (Statistical Analysing System) statistical software (SAS Institute Inc., 1982).

### Results

Eleven of our patients had their tumours located in the proximal part of the colon (caecum, ascending, transverse and descending) and 13 in the distal part (sigmoid and rectum). Six patients had tumours with a Dukes stage B, 14 with a stage C and four with a stage D. The majority of the tumours ($n = 14$) were of intermediate histological grade (grade 2), two tumours were well differentiated (grade 1) and eight were poorly differentiated tumours (grade 3) (Table I).

Plasma membrane enriched fractions of both colorectal carcinoma and normal colorectal mucosa biopsy samples were all ($n = 48$) found to contain EGFR (Table I). For the normal colorectal mucosa biopsy samples, the EGFR levels were significantly higher in samples from the proximal part of the colon than in samples from the distal part of the colon, the median EGFR levels being 124 fmol mg$^{-1}$ of membrane protein (range 70–239) and 55 fmol mg$^{-1}$ of membrane protein (range 35–156) $\chi^2_w = 4$, d.f. = 1, $P_w = 0.02$) (Figure 1a).

Carcinoma biopsy samples were shown to contain similar or decreased EGFR levels in 22/24 (92%) cases as compared with the respective normal mucosa samples, whereas in two rectal carcinomas, the EGFR levels were moderately higher. The EGFR levels in the normal colorectal mucosa biopsy samples (median EGFR level 77.5 fmol mg$^{-1}$ of membrane protein (range 35–239)) were significantly higher than the levels in the carcinoma biopsy samples (46 fmol mg$^{-1}$ of membrane protein (range 22–81)), irrespective of their intra-colic localisation (data not shown), both when analysed as paired observations ($t = 4.4$, d.f. = 1, $P_{t\text{-test}} = 0.001$), as well as when analysed as two separate groups ($\chi^2_w = 4$, d.f. = 1, $P_w = 0.001$) (Table I and Figure 2).

No regional variation in the EGFR levels of the colorectal carcinoma biopsy samples could be observed (Figure 1b). No association between the tumours EGFR content nor the normal/tumour EGFR ratio and patient age, sex, tumour stage or differentiation grade could be observed (data not shown).

**Table I** Patient and tumour characteristics

| No. | Age | Sex | Localisation | Stage | Grade | EGFR Mucosa | EGFR Carcinoma |
|---|---|---|---|---|---|---|---|
| 1 | 71 | M | Descending colon | D | 3 | 239 | 45 |
| 2 | 55 | M | Sigmoid | C | 3 | 47 | 24 |
| 3 | 69 | M | Rectum | B | 2 | 80 | 49 |
| 4 | 78 | M | Ascending colon | C | 2 | 70 | 22 |
| 5 | 54 | F | Rectum | C | 2 | 75 | 65 |
| 6 | 79 | F | Transverse colon | C | 2 | 80 | 58 |
| 7 | 73 | F | Sigmoid | B | 2 | 62 | 34 |
| 8 | 64 | M | Rectum | C | 3 | 53 | 37 |
| 9 | 84 | F | Caecum | B | 1 | 152 | 24 |
| 10 | 57 | F | Transverse colon | B | 2 | 156 | 23 |
| 11 | 86 | F | Ascending colon | D | 3 | 88 | 81 |
| 12 | 86 | F | Rectum | C | 2 | 55 | 45 |
| 13 | 59 | M | Caecum | D | 2 | 84 | 53 |
| 14 | 63 | F | Transverse colon | C | 2 | 134 | 34 |
| 15 | 71 | F | Rectum | C | 2 | 35 | 47 |
| 16 | 69 | F | Sigmoid | C | 2 | 71 | 32 |
| 17 | 53 | M | Rectum | C | 2 | 46 | 52 |
| 18 | 46 | M | Rectum | C | 3 | 46 | 31 |
| 19 | 60 | M | Rectum | C | 3 | 53 | 22 |
| 20 | 69 | M | Ascending colon | C | 2 | 124 | 64 |
| 21 | 57 | M | Rectum | B | 2 | 70 | 69 |
| 22 | 70 | M | Ascending colon | B | 3 | 91 | 77 |
| 23 | 59 | M | Caecum | D | 3 | 154 | 72 |
| 24 | 65 | M | Rectum | B | 1 | 156 | 49 |

Age: patient age in years; Sex: male, female patients; Stage: tumour stage according to Dukes. Grade: Histological tumour grade, 1 = well, 2 = moderately and 3 = poorly differentiated tumours. EGFR: fmol mg$^{-1}$ membrane protein.

**Figure 1** EGFR levels (expressed as fmol mg$^{-1}$ of membrane protein) found in normal **a**, and carcinomatous **b**, colorectal tissue biopsy samples according to their intra-colonic localisation: *C*aecum, *AS*cending colon, *TR*ansverse colon, *DES*cending colon, *SIG*moid and *REC*tum.

### Discussion

Our data, for the first time, clearly show that measurable amounts of EGFR are present in cell membrane preparations from colorectal carcinomas as well as in those from the normal colorectal mucosa. Moreover, EGFR levels were significantly higher in normal colorectal mucosa than in the corresponding carcinomas.

In previous studies employing EGFR ligand binding assays, other investigators did not find statistically significant differences in the EGFR levels between carcinomatous and normal colorectal tissue (Yasui *et al.*, 1988; Moorghen *et al.*, 1990; Rothbauer *et al.*, 1989), although in the study by Rothbauer *et al.* a trend towards higher EGFR levels in the normal colorectal mucosa, compared to the levels in the

**Figure 2** Relation between EGFR levels (expressed as fmol mg$^{-1}$ of membrane protein) found in human colorectal carcinoma tissue and the corresponding normal colorectal mucosa.

colorectal carcinomas was observed. We have shown (Figure 1a) that the inter-individual variation of EGFR levels in the normal colorectal mucosa has, at least in part, to be explained by topographical variations in EGFR levels. The use of (topographically) unpaired biopsy samples of colorectal mucosa and carcinoma in the study by Yasui *et al.* (1988) might therefore have obscured the difference between EGFR levels in normal and carcinomatous tissue. Second, in our study we exclusively isolated the colorectal mucosa by scraping it off the muscularis mucosae. Lacking information on the method of isolation of the normal colorectal mucosa by other groups, it is possible that the normal colorectal tissue samples they analysed comprised not only the mucosa, the tissue layer from which the colorectal carcinomas originate, but also the deeper colorectal tissue layers, shown to be devoid of EGFR (Zimmerman *et al.*, 1988). Should this have been the case, then they have underestimated, knowing that the EGFR levels are expressed as fmol mg$^{-1}$ of membrane protein, the EGFR levels in their normal colorectal 'mucosa' samples. Third, as ligand binding assays are apt to give a false negative assay result if the membrane protein level falls below a certain threshold (0.2 mg of membrane protein ml$^{-1}$ when assaying breast carcinomas, Koenders *et al.*, 1991) the substantial amount (0.5 mg of membrane protein ml$^{-1}$) of colorectal tissue cell membrane protein used in our series contributes to the validity of our results. The use of a multiple point EGFR assay in our series might explain the higher levels of EGFR obtained. In the previous studies using ligand binding assays a total of 80 colorectal tissue samples, carcinoma or normal tissue, were analysed, and in all cases the presence of EGFR could be demonstrated (Yasui *et al.*, 1988; Rothbauer *et al.*, 1989; Moorghen *et al.*, 1990). In sharp contrast with these observations Magnusson *et al.*, 1989 also employing a ligand binding assay, reported EGFR in only 25% of colorectal cancers. Studies using immunohistochemical methods for the detection of EGFR reported percentages of EGFR positivity varying from 25% to 100% in colorectal carcinomas (Ravikumar *et al.*, 1989; Steele *et al.*, 1990*b*) and from 0% to 50% in normal colorectal tissue (Ravikumar *et al.*, 1989; Koretz *et al.*, 1990). Thus, except for one study, results on the prevalence of EGFR, using ligand binding assays, are consistent in that both colorectal carcinomas and normal colorectal tissue contain detectable levels of EGFR, whereas the percentages of EGFR positivity reported by groups using immunohistochemical methods to detect EGFR are at variance with each other, but lower than those reported by groups using ligand binding assays.

The presence of high EGFR levels in the normal colorectal mucosa along with the observed regional differences in colorectal EGFR levels suggest EGFR to be implicated in the process of growth and differentiation of the normal colorectal mucosa. The clinical implementation of EGFR and/or EGF targeted anti-cancer drugs, might therefore be seriously impaired, due to the anticipated growth inhibitory effects of these drugs on the intestinal mucosa.

Our observation that EGFR is significantly lower in colorectal carcinomas than in the respective normal colorectal mucosa indicates that the original EGFR content decreases upon malignant transformation. In contrast, Liu *et al.*, 1990 reported that TGF$\alpha$ is expressed at higher levels in colorectal carcinomas than in normal colorectal tissue, it may therefore well be that the lower EGFR content in colorectal carcinomas is caused by a downregulation of the receptor by a locally produced ligand. Therefore the tumours capability to produce growth factors rather than the expression of the growth factor receptor might be the factor determining its growth capacity.

### References

ANZANO, M.A., RIEMAN, D., PRICHETT, W., BOWEN-POPE, D.F. & GREIG, R. (1989). Growth factor production by human colon carcinoma cell lines. *Cancer Res.*, **49**, 2898.

ASTLER, W.B. & COLLER, F. (1954). The prognostic significance of direct extension of carcinoma of the colon and rectum. *Ann. Surg.*, **139**, 846.

BENRAAD, Th.J. & FOEKENS, J.A. (1990). Hydroxyapatite assay to measure epidermal growth factor receptor in human primary breast tumors. *Ann. Clin. Biochem.*, **27**, 272.

BRADLEY, S.J., GARFINKLE, G., WALKER, E., SALEM, R., CHEN, L.B. & STEELE, G. (1986). Increased expression of the epidermal growth factor receptor on human colon carcinoma cells. *Arch. Surg.*, **121**, 1242.

COFFEY, R.J., GOUSTIN, A.S., MANGELSDORF SODERQUIST, A. & 4 others (1987). Transforming growth factor $\alpha$ and $\beta$ expression in human colon cancer lines: implications for an autocrine model. *Cancer Res.*, **47**, 4590.

HANAUSKE, A.R., BUCHOK, J., SCHEITHAUER, W. & VON HOFF, D.D. (1987). Human colon cancer cell line secrete $\alpha$ TGF-like activity. *Br. J. Cancer*, **55**, 57.

KOENDERS, P.G., BEEX, L.V.A.M., GEURTS-MOESPOT, A., HEUVEL, J.J.T.M., KIENHUIS, C.B.M. & BENRAAD, Th.J. (1991). Epidermal growth factor receptor negative tumors are predominantly confined to the subgroup of estradiol receptor positive human primary breast cancers. *Cancer Res.*, **51**, 4544.

KORETZ, K., SCHLAG, P. & MÖLLER, P. (1990). Expression of epidermal growth factor receptor in normal colorectal mucosa, adenoma and carcinoma. *Virchows Archiv. A Pathol. Anat.*, **416**, 343.

LIU, C., WOO, A. & TSAO, M.S. (1990). Expression of transforming growth factor-alpha in primary human colon and lung carcinomas. *Br. J. Cancer*, **62**, 425.

LOWRY, O.H., ROSEBROUGH, N.J., FARR, A.L. & RANDALL, R.J. (1951). Protein measurement with the Folin phenol reagent. *J. Biol. Chem.*, **193**, 265.

MAGNUSSON, I., ROSEN, A.V., NILSSON, R., MACIAS, A., PEREZ, R. & SKOOG, L. (1989). Receptors for epidermal growth factor and sex steroid hormones in human colorectal carcinomas. *Anticancer Res.*, **9**, 299.

MOORGHEN, M., INCE, P., FINNEY, K.J., WATSON, A.J. & HARRIS, A.L. (1990). Epidermal growth factor receptors in colorectal carcinomas. *Anticancer Res.*, **10**, 605.

MURTHY, U., ANZANO, M.A. & GREIG, R.G. (1989). Expression of TGF$\alpha$/EGF and TGF$\beta$ receptors in human colon carcinoma cell lines. *Int. J. Cancer*, **44**, 110.

RAVIKUMAR, T.S., WOLF, B., COCCHIARO, ?., D'EMILIA, J. & STEELE, G. (1989). RAS gene activation and epidermal growth factor receptor expression in human colon cancer. *J. Surg. Res.*, **47**, 418.

ROTHBAUER, E., MANN, K., WIEBECKE, B. & 5 others (1989). Epidermal growth factor receptors and epidermal growth factor-like activity in colorectal mucosa, adenomas and carcinomas. *Klin. Wochenschrift*, **67**, 518.

SAS (STATISTICAL ANALYZING SYSTEM) INSTITUTE INC. (1982). *User's Guide: Statistics*, verson 5 edition, SAS Institute Inc, Box 8000, Cary, NC 27511-8000.

SCATCHARD, G. (1949). The attraction of proteins for small molecules and ions. *Ann. NY Acad. Sci.*, **51**, 660.

SPORN, M.B. & TODARO, G.J. (1980). Autocrine secretion and malignant transformation of cells. *N. Engl. J. Med.*, **303**, 878.

SPORN, M.B. & ROBERTS, A.B. (1985). Autocrine growth factors and cancer. *Nature*, **313**, 745.

STEELE, R.J.C., KELLY, P., ELLUL, B. & EREMIN, O. (1990*a*). Immunohistochemical detection of epidermal growth factor receptors on human colonic carcinomas. *Br. J. Cancer*, **61**, 325.

STEELE, R.J.C., KELLY, P., ELLUL, B. & EREMIN, O. (1990*b*). Epidermal growth factor receptor expression in colorectal cancer. *Br. J. Surg.*, **77**, 1352.

YASUI, W., SUMIYOSHI, H., HATA, J. & 4 others (1988). Expression of epidermal growth factor receptor in human gastric and colonic carcinomas. *Cancer Res.*, **48**, 137.

ZIMMERMAN, R.P., GATES, T.S., BOEHMER, C.G. & MANTYH, P.W. (1988). Epidermal growth factor receptors in the human colon. *Eur. J. Pharmacol.*, **150**, 201.